# Supplementary material for: Integrative Korean Medicine Treatments for Traumatic Facial Palsy Following Mandibular Fracture: A Case Report and Literature Review
Source: Healthcare (Basel). 2023 Sep 14;11(18):2546. doi: 10.3390/healthcare11182546 (PMC10530994; doi:10.3390/healthcare11182546)

Supplementary Table S1.

Search strategy in PubMed - 230627

<table>
<tr><th></th><th>Search strategy</th><th>Item</th></tr>
<tr><td>#1.</td><td>(Traumatic Facial Palsy[TW]) OR (Traumatic Facial Paralysis[TW]) OR (Traumatic Facial Nerve Palsy[TW]) OR (Traumatic Facial Nerve Paralysis[TW])</td><td>200</td></tr>
<tr><td>#2</td><td>(Korean Medicine[TW])|(Oriental Medicine[TW])|(Chinese Medicine[TW])|(Traditional Medicine[TW])|(Traditional Korean Medicine[TW])|(Traditional Chinese Medicine[TW])|(Traditional Oriental Medicine[TW])|(Acupuncture[TW])|(Herbal Medicine[TW])</td><td>111,328</td></tr>
<tr><td>#3</td><td>#1 AND #2</td><td>3</td></tr>
</table>

Search strategy in Cochrane library - 230627

	Search strategy	Item
#1	(Traumatic facial palsy):ti,ab,kw OR (Traumatic facial paralysis):ti,ab,kw OR (Traumatic facial nerve palsy):ti,ab,kw OR (Traumatic facial nerve paralysis):ti,ab,kw	10
#2	(Korean Medicine):ti,ab,kw OR (Oriental Medicine):ti,ab,kw OR (Chinese Medicine):ti,ab,kw OR (Traditional Medicine):ti,ab,kw OR (Traditional Korean Medicine):ti,ab,kw OR (Traditional Chinese Medicine):ti,ab,kw OR (Traditional Oriental Medicine):ti,ab,kw OR (Acupuncture):ti,ab,kw OR (Herbal Medicine):ti,ab,kw	35,903
#3	#1 AND #2	3

Search strategy in Embase - 230627

	Search strategy	Item
#1	(Traumatic facial palsy):ti,ab,kw OR (Traumatic facial paralysis):ti,ab,kw OR (Traumatic facial nerve palsy):ti,ab,kw OR (Traumatic facial nerve paralysis):ti,ab,kw	669
#2	(Korean Medicine):ti,ab,kw OR (Oriental Medicine):ti,ab,kw OR (Chinese Medicine):ti,ab,kw OR (Traditional Medicine):ti,ab,kw OR (Traditional Korean Medicine):ti,ab,kw OR (Traditional Chinese Medicine):ti,ab,kw OR (Traditional Oriental Medicine):ti,ab,kw OR (Acupuncture):ti,ab,kw OR (Herbal Medicine):ti,ab,kw	174,135
#3	#1 AND #2	8

Search Strategy in CNKI - 230627

	Search strategy	Item
#1	SU=(外伤性面瘫) OR SU=(创伤性面瘫) OR SU=(外伤性面神经麻痹) OR SU=(创伤性面神经麻痹) OR SU=(traumatic facial palsy) OR SU=(traumatic facial paralysis) OR SU=(traumatic facial nerve palsy) OR SU=(traumatic facial nerve paralysis)	174
#2	SU=(中医) OR SU=(中药) OR SU=(针) OR SU=(针刺) OR SU=(Oriental Medicine) OR SU=(Chinese Medicine) OR SU=(Traditional Medicine) OR SU=(Traditional Chinese Medicine) OR SU=(Traditional Oriental Medicine) OR SU=(Herbal Medicine) OR SU=(Acupuncture)	1,329,933
#3	#1 AND #2<br>(SU=(外伤性面瘫) OR SU=(创伤性面瘫) OR SU=(外伤性面神经麻痹) OR SU=(创伤性面神经麻痹) OR SU=(traumatic facial palsy) OR SU=(traumatic facial paralysis) OR SU=(traumatic facial nerve palsy) OR SU=(traumatic facial nerve paralysis)) AND (SU=(中医) OR SU=(中	25

药) OR SU=(针) OR SU=(针刺) OR SU=(Oriental Medicine) OR SU=(Chinese Medicine) OR SU=(Traditional Medicine) OR SU=(Traditional Chinese Medicine) OR SU=(Traditional Oriental Medicine) OR SU=(Herbal Medicine) OR SU=(Acupuncture))

Search Strategy in RISS - 230628

	Search strategy	Item
#1	전체 = 한의 l 한방 l 한의치료 l 한방치료 l 침 l 한약	38,753
#2	전체 = (Korean Medicine) l (Oriental Medicine) l (Traditional Medicine) l (Traditional Korean Medicine) l (Traditional Oriental Medicine) l Acupuncture l (Herbal Medicine)	46,165
#3	#1 OR #2	75,875
#4	전체 = (외상성 안면마비) l (외상성 구안와사)	15
#5	전체 = (Traumatic facial palsy) l (Traumatic facial paralysis) l (Traumatic facial nerve palsy) l (Traumatic facial nerve paralysis)	59
#6	#4 OR #5	64
#7	#3 AND #6	11

Search Strategy in KISS - 230628

	Search strategy	Item
#1	전체 = 한의 l 한방 l 한의치료 l 한방치료 l 침 l 한약	351,706
#2	전체 = (Korean Medicine) l (Oriental Medicine) l (Traditional Medicine) l (Traditional Korean Medicine) l (Traditional Oriental Medicine) l Acupuncture l (Herbal Medicine)	71,681
#3	#1 OR #2	406,484
#4	전체 = (외상성 안면마비) l (외상성 구안와사)	7
#5	전체 = (Traumatic facial palsy) l (Traumatic facial paralysis) l (Traumatic facial nerve palsy) l (Traumatic facial nerve paralysis)	34
#6	#4 OR #5	35
#7	#3 AND #6	10

Search Strategy in OASIS - 230628

	Search strategy	Item
#1	전체 = 한의 l 한방 l 한의치료 l 한방치료 l 침 l 한약	4,261
#2	전체 = (Korean Medicine) l (Oriental Medicine) l (Traditional Medicine) l (Traditional Korean Medicine) l (Traditional Oriental Medicine) l Acupuncture l (Herbal Medicine)	9,179
#3	#1 OR #2	12,728
#4	전체 = (외상성 안면마비) l (외상성 구안와사)	3
#5	전체 = (Traumatic facial palsy) l (Traumatic facial paralysis) l (Traumatic facial nerve palsy) l (Traumatic facial nerve paralysis)	4
#6	#4 OR #5	6
#7	#3 AND #6	6

Search Strategy in ScienceOn - 230628

	Search strategy	Item
#1	전체 = 한의 l 한방 l 한의치료 l 한방치료 l 침 l 한약	65,027

#2	전체 = (Korean Medicine) | (Oriental Medicine) | (Traditional Medicine) | (Traditional Korean Medicine) | (Traditional Oriental Medicine) | Acupuncture | (Herbal Medicine)	377,100
#3	#1 OR #2	389,930
#4	전체 = (외상성 안면마비) | (외상성 구안와사)	21
#5	전체 = (Traumatic facial palsy) | (Traumatic facial paralysis) | (Traumatic facial nerve palsy) | (Traumatic facial nerve paralysis)	46
#6	#4 OR #5	55
#7	#3 AND #6	15

Supplementary Table S2. Characteristics of included case studies on traumatic facial palsy.

Author (Y)	Study Design	Participant Characteristics				Treatment Method(s)		Outcome Measurements	AE(s)
		Cases of Traumatic Facial Palsy	Sample Size (N)	Male/ Female (N)	Age (year) Mean ± SD or Median or (minimum to maximum)	Intervention group (N)	Control group (N)		
Wang (2022) [1]	Case report	Fractures of the temporal bones, occipital bone, and right frontal bone.	1	0/1	2	Electroacupuncture + Hyperbaric oxygen + Conventional medication (methylprednisolone)		HB grade	N.R
Ton (2019) [2]	Case report	Traumatic brain contusion	1	1/0	52	Laser acupuncture		1) FDI<br>2) SB<br>3) VPD<br>4) Photograph	N.R
Jang (2016) [3]	Case report	Temporal Bone Fx.	1	0/1	19	Acupuncture + Pharmacopuncture + Herbal medicine + Physiotherapy + Conventional medication		1) HB grade<br>2) Yanagihara's point<br>3) Digital Infrared Thermographic Image	N.R
Liang (2014) [4]	Case Series	Basal skull fx.	23	16/7	17–68	Electroacupuncture		TER	N.R
Xu (2013) [5]	Case Series	Head trauma	45	32/13	8–72	Acupuncture + Electroacupuncture + Scalp acupuncture		TER	N.R
Ge (2010) [6]	Case Series	Craniocerebral Injury	76	56/20	36.5 (16–78)	Acupuncture + Herbal medicine + Conventional medication + HBOT		TER	N.R
Liu (2009) [7]	Case Series	Skull fx.	32	22/10	42	Electroacupuncture + Moxibustion		TER	N.R

Xu (2009) [8]	Case Series	Temporal Bone Fx.	13	9/4	34.2 (18–48)	Acupuncture + TDP irradiation therapy + Po Medication	TER	N.R
Zhang (2009) [9]	Case Series	Head Trauma	31	21/10	8–50	Scalp acupuncture + Conventional medication injection (Mecobal injection)	TER	N.R
Zhang (2008) [10]	Case Series	Skull fx.	50	36/14	10–54	Warm needle acupuncture + Electroacupuncture + Conventional medication (Vit. B12) injection	TER	N.R
Ahn (2007) [11]	Case report	Temporal Bone Fx.	1	1/0	54	Acupuncture + Herbal medicine + Electroacupuncture + Physiotherapy + Moxa hot pack	1) HB grade<br>2) Yanagihara's point<br>3) DITI<br>4) EMG	N.R
Xing (2007) [12]	Case Series	N.R	30	18/12	38 (22–48)	Cupping + Fire acupuncture + Blood letting	TER	N.R
Lee (2006) [13]	Case Series	Head Trauma with Lt Temporal Bone Fx, Lt External Ear Fx	2	1/1	1) 34<br>2) 2	Acupuncture+ Herbal medicine + Physiotherapy + Facial Muscle Exercise	Yanagihara's point	N.R
Yang (2006) [14]	Case Series	Skull fx.	30	22/8	5–56	Acupuncture + Moxibustion + HBOT	TER	N.R
Gu (2005) [15]	Case Series	Head trauma	59	40/19	6–53	Acupuncture	TER	N.R
Zhou (2005) 16]	Case Series	Craniocerebral injury	15	15/0	37 (5–49)	Electroacupuncture + Laser acupuncture + Conventional medication (dibazole, Multi Vitamin B) + Herbal medicine	TER	N.R
Kim (2003) [17]	Case report	Skull Fx with SDH	1	1/0	9	Herbal medicine + Acupuncture + Electroacupuncture + Physiotherapy + Cupping + Moxa hot pack	HB grade	N.R

Choi (2002) [18]	Case report	Temporal Bone Fx.	1	1/0	36	Acupuncture + Pharmacopuncture + Herbal medicine + Cupping + Physiotherapy	1) HB grade<br>2) Yanagihara's point<br>3) DITI<br>4) ENoG	N.R
Zhu (2002) [19]	Case Series	Fx. of the skull (base)	36	27/9	12–67	Acupuncture + Scalp acupuncture + Warm needle acupuncture	TER	N.R
Li (2001) [20]	Case Series	Fx. of the skull base, Facial fx., Contusions of facial soft tissue	60	N.R	N.R	Scalp acupuncture + Warm needle acupuncture	TER	N.R
Li (2001) [21]	Case Series	Operation side effect, Nerve injury by trauma	2	2/0	1) 34<br>2) 43	Acupuncture + Electroacupuncture	N.R	N.R
Zhou (2001) [22]	Case Series	Craniocerebral trauma (Skull fx. 9)	12	7/5	29.6 (15–53)	Acupuncture + Herbal medicine (Boyanghwanoh-tang) (Local massage + Moxibustion + Functional exercises)	TER	N.R
Wang (2000) [23]	Case Series	Middle skull base Fx.	29	16/13	6–49	Acupuncture	TER	N.R
Liu (1994) [24]	Case Series	N.R	48	31/17	15–55	Scalp acupuncture	TER	N.R
Li (1984) [25]	Case report	Head trauma	1	0/1	57	Acupuncture	N.R	N.R

AEs: Adverse Events; DITI: Digital Infrared Thermographic Image; EMG: Electromyography; ENoG: Electroneurography; FDI: Facial Disability Index; Fx.: Fracture; HB grade: House–Brackmann Grade; HBOT: Hyperbaric Oxygen Therapy; N.R: Not Reported; RCT: Randomized Controlled Trial; SB: Sunnybrook Facial Grading System; SDH: subdural hemorrhage; TER: Total Effective Rate.

References

1. Wang, L.; Shi, H. Treatment of traumatic facial paralysis in a child with electroacupuncture and hyperbaric oxygen: A case report. Complement Ther Clin Pract. 2022, 48, 101595, doi:10.1016/j.ctcp.2022.101595.
2. Ton, G.; Lee, L.W.; Chen, Y.H.; Tu, C.H.; Lee, Y.C. Effects of laser acupuncture in a patient with a 12-year history of facial paralysis: A case report. Complement Ther Med. 2019, 43, 306-310, doi:10.1016/j.ctim.2019.02.015.
3. Jang, Y.J.; Yang, T.J.; Shin, J.C.; Kim, H.H.; Kim, T.G.; Jeong, M.Y., et al. Clinical Case Study of Facial Nerve Paralysis with Sensorineural Hearing Loss and Tinnitus Caused by Traumatic Temporal Bone Fracture. J. Acupunct Res. 2016, 33, 95-101, doi:10.13045/acupunct.2016009.
4. Liang, Y. Clinical observation on the treatment of 23 cases with traumatic facial paralysis by electro-acupuncture. Clin. J. Chin. Med. 2014, 6, 45-46, doi:CNKI:SUN:ZYLY.0.2014-27-024.
5. Xu, H. Acupuncture treatment for 45 cases of traumatic facial paralysis. Hunan. J. Tradit. Chin. Med. 2013, 29, 75, doi:10.16808/j.cnki.issn1003-7705.2013.08.041.
6. Ge, F.; Xu, R.; Tang, Z.F., et al. Study of Acupuncture TCM and Western Medicine and Hyperbaric Oxygen Integrative Therapy in Treatment of Facial Paralysis Induced by Craniocerebral Injury. J. Liaoning Univ. Tradit. Chin. Med. 2010, 12, 170-171, doi:10.13194/j.jlunivtcm.2010.12.172.gef.087.
7. Liu, J.H. Treatment of 32 Cases of Traumatic Facial Paralysis with Electroacupuncture and Ginger-separated Moxibustion. J. External Ther. Tradit. Chin. Med. 2009, 18, 38, doi:CNKI:SUN:ZYWZ.0.2009-06-032.
8. Xu, W. Treatment of Facial Paralysis Caused by Temporal Bone Fracture with Acupuncture as the Main Therapy. Zhejiang J. Integr. Tradit. Chin. West. Med. 2009, 19, 381, doi:CNKI:SUN:ZJZH.0.2009-06-037.
9. Zhang, Z.Y.; Zhang, Q.L. Treatment of 31 Cases of Traumatic Facial Paralysis with Acupuncture. Shanghai J. Acupunct. Moxibustion. 2009, 28, 662, doi:CNKI:SUN:SHZJ.0.2009-11-032.
10. Zhang, B.Q. Treatment of 50 Cases of Traumatic Facial Paralysis with Warm Needle Moxibustion Combined with Acupoint Injection. China Mod. Dr. 2008, 96-97, doi:CNKI:SUN:ZDYS.0.2008-07-057.
11. Ahn, H.L.; Shin, M.S. A Case Report of a Patient with Facial Nerve Paralysis Caused by Traumatic Temporal Bone Fracture. J Korean Med Rehabi. 2007, 17, 159-166.
12. Xing, L.L.; Chen, Z. Observation on the Efficacy of San Tong Method in the Treatment of 30 Cases of Traumatic Facial Paralysis. Asia-Pacific Tradit. Med. 2007, 56-57, doi:CNKI:SUN:YTCT.0.2007-05-026.
13. Lee, J.M.; Kim, E.M.; Song, H.G.; Go, S.K.; Kim, S.L.; Kim, J.H., et al. Clinical Study of Two Patients with Deviation of the Eye and Mouth Caused by Trauma. J. Acupunct Res. 2006, 23, 81-89.
14. Yang, S.X.; Cai, Y. Treatment of 30 Cases of Traumatic Facial Paralysis with Acupuncture Combined with Hyperbaric Oxygen. Shaanxi J. Tradit. Chin. Med. 2006, 1569-1570, doi:CNKI:SUN:SXZY.0.2006-12-079.
15. Gu, W. Clinical observation of acupuncture treatment for 59 cases of traumatic facial paralysis. J. Clin. Acupunct. Moxibustion. 2005, 8, doi:CNKI:SUN:ZJLC.0.2005-07-006.
16. Zhou, L.Z.; Huang, Y.; Ao, J.B., et al. Treatment of 15 Cases of Traumatic Facial Paralysis with Acupuncture as the Main Therapy. Chin. J. Phys. Med. Rehabil. 2005, 61, doi:CNKI:SUN:ZHLY.0.2005-02-00Q.
17. Kim, K.H.; Shin, D.G.; Kim, D.G. A Case of Facial Palsy and Hearing Disturbance Caused by Traumatic Disorder. J Korean Oriental Pediat-rics. 2003, 17, 137-148.
18. Choi, S.W.; Roh, J.D.; Sin, M.S.; Seol, H.; Song, B.Y.; Yook, T.H. Clinical Study of Patient with Facial Nerve Paralysis Caused by Traumatic Temporal Bone Fracture. J. Acupunct Res. 2002, 19, 207-215.
19. Zhu, C.Y. Treatment of 36 Cases of Traumatic Facial Paralysis with Acupuncture. Chin. Acupunct. Moxibustion. 2002, 31, doi:CNKI:SUN:ZGZE.0.2002-09-014.
20. Li, S.C. Observation on the Curative Efficacy of Acupuncture combined with Warm Needle Moxibustion for the Treatment of 60 Cases of Traumatic Facial Paralysis. New J. Tradit. Chin. Med. 2001(04), 45, doi:10.13457/j.cnki.jncm.2001.04.027.
21. Li, B.H.; Wu, Z.Q.; Qi, W. Report on 2 Cases of Traumatic Facial Paralysis. J. Tradit. Chin. Med. Chin. Mater. Med. Jilin. 2001, 7, doi:10.13463/j.cnki.jlzyy.2001.05.006.
22. Zhou, Y.; Zheng, Z.S.; Yang, B. Treatment of 12 Cases of Traumatic Facial Paralysis with Acupuncture and Herbal Medicine. Shandong J. Tradit. Chin. Med. 2001, 480-481, doi:CNKI:SUN:SDZY.0.2001-08-021.
23. Wang, L.; Yang, C.G.; Liu, H.L. Clinical Observation on the Treatment of 29 Cases of Facial Paralysis with Acupuncture. Chin. Acupunct. Moxibustion. 2000, 21-22, doi:10.13703/j.0255-2930.2000.08.014.
24. Liu, F.T.; Zhang, C.C.; Yu, Y.H. Treatment of 48 Cases of Traumatic Facial Paralysis Primarily with Otopoint Therapy. Shanghai J. Acupunct. Moxibustion. 1994, 166, doi:10.13460/j.issn.1005-0957.1994.04.015.
25. Li, G. Acupuncture cure for a case of traumatic facial paralysis with locked jaw. Shanghai J. Acupunct. Moxibustion. 1984, 8, doi:10.13460/j.issn.1005-0957.1984.02.005.

Supplementary Figure S1. Flowchart of the studies selection process.

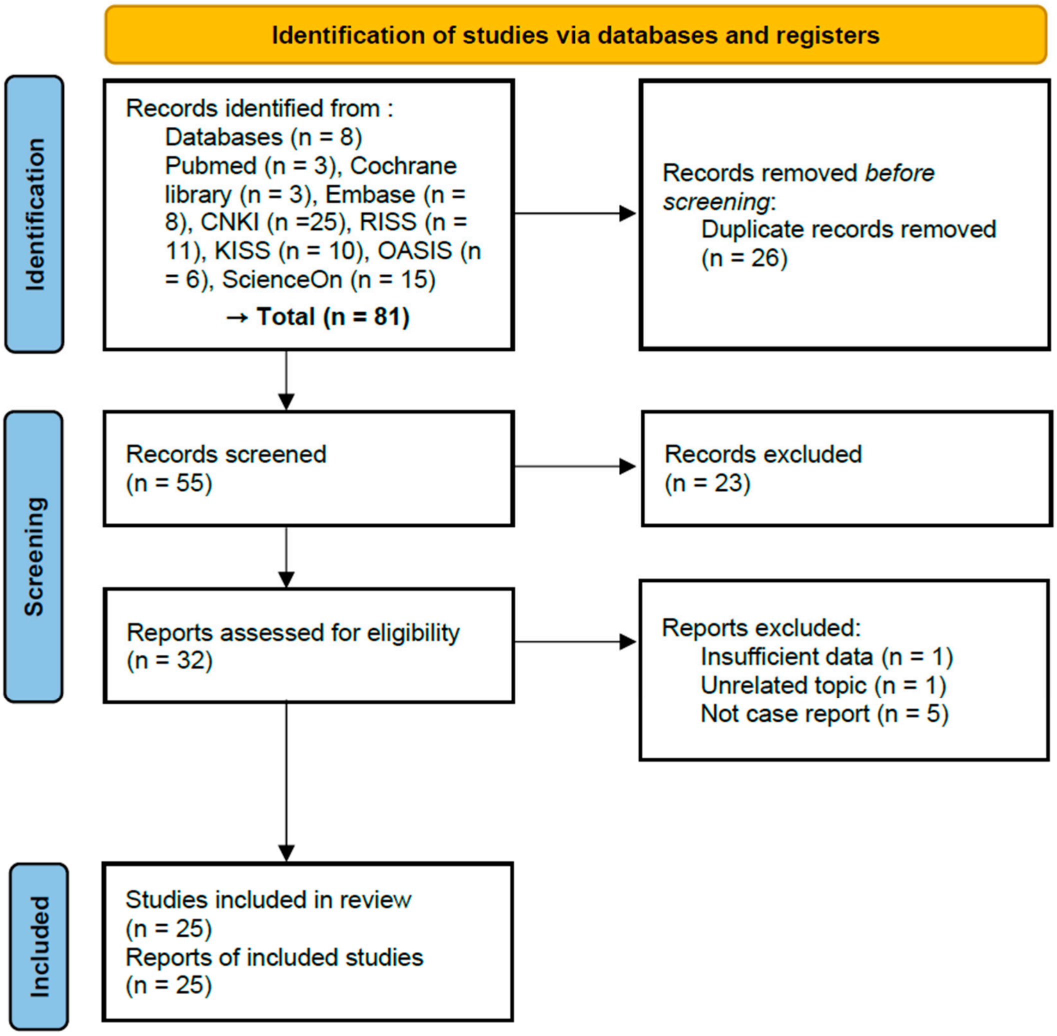

Supplement: Supplementary file 1 [file healthcare-11-02546-s001.zip › healthcare-2567644-supplementary.pdf]
